# Semantic web data warehousing for caGrid

**DOI:** 10.1186/1471-2105-10-S10-S2

**Published:** 2009-10-01

**Authors:** Jamie P McCusker, Joshua A Phillips, Alejandra González Beltrán, Anthony Finkelstein, Michael Krauthammer

**Affiliations:** 1 Department of Pathology, Yale University School of Medicine, New Haven, CT USA; 2 Semantic Bits, LLC, Reston, VA USA; 3 Department of Computer Science, University College London, London, UK

**Keywords:** Unify Modeling Language, Resource Description Framework, Grid Service, Unify Modeling Language Model, Service Metadata

## Abstract

The National Cancer Institute (NCI) is developing caGrid as a means for sharing cancer-related data and services. As more data sets become available on caGrid, we need effective ways of accessing and integrating this information. Although the data models exposed on caGrid are semantically well annotated, it is currently up to the caGrid client to infer relationships between the different models and their classes. In this paper, we present a Semantic Web-based data warehouse (Corvus) for creating relationships among caGrid models. This is accomplished through the transformation of semantically-annotated caBIG^®^ Unified Modeling Language (UML) information models into Web Ontology Language (OWL) ontologies that preserve those semantics. We demonstrate the validity of the approach by Semantic Extraction, Transformation and Loading (SETL) of data from two caGrid data sources, caTissue and caArray, as well as alignment and query of those sources in Corvus. We argue that semantic integration is necessary for integration of data from distributed web services and that Corvus is a useful way of accomplishing this. Our approach is generalizable and of broad utility to researchers facing similar integration challenges.

**Electronic supplementary material:**

The online version of this article (doi:10.1186/1471-2105-10-S10-S2) contains supplementary material, which is available to authorized users.

## Introduction and background

We propose a Semantic Web data warehouse approach that enables users to map data from multiple grid data sources into an ontologically-driven data store, or knowledge base (KB), where they can use data from a semantic perspective. caGrid, a core technology of caBIG^®^ ("Cancer Biomedical Informatics Grid") [1–5], is a semantically annotated grid sponsored by the National Cancer Institute that provides a consistent framework for grid web services. The information models of the grid services are mapped to concepts from the NCI Thesaurus (NCIt) [6–9], a rich, cancer-focused terminology source, through Common Data Elements (CDEs) registered in the Cancer Data Standards Repository (caDSR) [10]. The grid services advertise the information models that they support to a centralized Index Service for use by grid clients. CDEs represent semantically interoperable "join points" among information models, which provide a basis for data integration.

Clients access caGrid to retrieve data from diverse services such as *omic* [13] stores (caArray) or tissue repositories (caTissue). From the client perspective, there is no transparent mapping of semantics onto data from grid services. When a caGrid client wants to join data from one service to another, or attempts to make claims about a particular datum being equivalent to another, it must inspect the metadata to determine if, and how, data from two services are interoperable. A naive client that is unaware of the service metadata will be unable to make that mapping. In other words, semantic interoperability is the job of the client and requires the ability to reason over (or interpret) the metadata, including class hierarchies, attributes, associations, and their corresponding annotations to establish equivalencies.

Fortunately, there is already a solution that is available that can perform exactly those tasks: The Web Ontology Language (OWL) [14] is a formal way of describing relationships among concepts and any data defined in the Resource Description Framework (RDF) [15, 16]. OWL is relevant here because it provides for class hierarchies, properties, and equivalencies. It also provides a means for multiple ontologies to coexist and for mappings to be defined between them. A client that can take advantage of the formal definitions of OWL through inferencing rules would have the ability to automatically map between data models on the grid. We show that a client that imports data from multiple grid services and maps that data onto ontologies derived from the published service metadata could then join that data within the Semantic Web environment to allow a much larger set of queries to be realized.

Semantic Web data warehousing allows users to define which data sources they are interested in and automates the extraction, transformation, and loading process (ETL) through semantic ETL (SETL) [17] across entire classes of data sources. Semantic Web data warehouses are dynamic data stores, which, as we will show, can model and store data from diverse grid services on the fly. Users will be able to query the grid in novel ways using the data warehouse as a proxy and will be able to dynamically integrate new data sources as needed.

### Transforming UML to OWL

In order to perform semantic-web-based SETL on caGrid services, it is imperative to understand how to map UML constructs and their NCIt annotations (caGrid models) onto semantic-web constructs (OWL ontologies). UML is the *de facto* standard for object-oriented visual modeling and has no formal semantics. Its main constructs are classes, attributes, associations, and generalizations. A UML class is a representation of an object-oriented class, which is defines a set of objects with common characteristics indicated as attributes. An association is a relation between classes and a generalization relates a parent class with a child class. On the other hand, OWL is a knowledge modeling language with a formal semantics based on Description Logics (DLs) [18]. Its main constructs are classes, datatype properties, and object properties. An OWL class denotes a set of individuals or instances. Properties are standalone entities, establishing relationships between individuals (object properties) or between individuals and data values (datatype properties) [14]. Although UML and OWL have similar constructs, they have significant differences. Mainly, UML follows a Closed World Assumption (CWA) while OWL follows an Open World Assumption (OWA). In CWA, lack of information means negative information. In OWA, lack of information means lack of knowledge.

Previous work has compared and contrasted UML and OWL and provided transformations between the two [19–25]. These transformations were motivated by different applications and specified in varying levels of detail. For example, Berardi *et al* [19] provided an incomplete transformation from UML class diagrams to description logics and analyzed the complexity of the reasoning to detect inconsistencies in the model. Evermann [23] described an exhaustive conversion to make a well-known ontology, specified in natural language, available in more formal representations.

To the best of our knowledge, semCDI [24, 25] is the only work providing an annotated-UML-to-OWL transformation based on the caGrid infrastructure. semCDI, as with all the previous approaches, maps UML classes to OWL classes, UML attributes to datatype properties, and associations to object properties. caGrid UML classes are annotated with NCIt concepts, and there is a need to represent these associations in OWL. semCDI does this by creating parent-child relationships between the OWL-converted UML class (the child) and the associated NCIt class (the parent), using concepts of an OWL-formatted NCIt. Using subsumption to represent this relationship results in a potentially inconsistent ontology. Examples include situations where a UML class is annotated with two or more NCIt concepts, some of which are explicitly stated as disjoint in NCIt. In caGrid, attributes are also annotated with NCIt concepts. As semCDI represents attributes as datatype properties, there are some difficulties in representing the NCIt associations. The only available option is to represent the NCIt annotation as an OWL annotation property. However, OWL annotation properties are used to represent metadata on OWL constructs and are not considered for reasoning purposes.

Considering the issues presented above, we have designed a different annotated UML-to-OWL transformation that does not model attributes as datatype properties and does not model NCIt annotations of UML classes using subsumption. Our transformation, described in detail under the Methods section, follows a general, modular approach for ontology development. Particularly, it includes a common approach for modeling NCIt annotation for both UML classes and attributes, which guarantees to preserve NCIt semantics.

### Methods overview

In order to assess the feasibility of Semantic Web-based SETL on caGrid services, we first identified a real-world use case involving caGrid that included the need for semantically merging disparate information models. Specifically, we identified the need to join data from caTissue and caArray, two caGrid services exposing tissue and micro-array data, respectively. The use case involves the need to link a microarray experiment with clinical annotations linked to the specimen from which the experiment was derived. Imagine a situation where a specimen *S* is stored in caTissue and a microarray result *M* (derived from specimen *S*) is stored in caArray. Currently, it is possible to query caGrid for a single service using the caGrid Query language (CQL). Assume that we get results (data) from caTissue on *S*, which we call *R*_*s*_. Equally, we get results from querying caArray, which we call *R*_*m*_. As discussed above, the linking of *R*_*s*_to *R*_*m*_is not trivial. There is a need to identify the classes and attributes in both the caTissue and caArray models that align and the constraints under which two instances from the two models can be linked together.

In this paper, we demonstrate that this can be elegantly accomplished using Semantic Web technology. We first set up an instance of caTissue and caArray on the caGrid training grid. We then loaded specimen information of a particular set of cell lines called NCI-60 [26] into caTissue. NCI-60 is a collection of cancer cell lines for which there exists a multitude of micro-array experiments (gene expression or copy number experiments). We recorded the disease class of each of the cell lines in caTissue. We then loaded an NCI-60 gene expression set into our caArray instance. The quest was to link the caTissue and caArray datasets, i.e. link the expression sets in caArray with specific disease information in caTissue. We will present how we use SETL to perform this linking.

## Results and discussion

**Figure 1:**
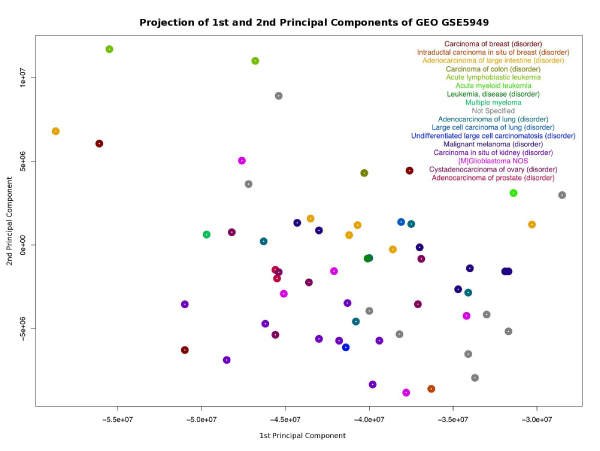
**Principal components analysis**. Projection of the first two principal components of gene expression microarray experiment GSE5949 from GEO. The clinical diagnoses for the biological source cell line were extracted from caTissue and joined using Corvus.

We were able to successfully load data from caTissue and caArray into a Semantic Web data repository, which we call Corvus, and link the instances of the caArray *Source* class with the instances of the caTissue *CellSpecimen* class, allowing us to use clinical data from caTissue to enhance the analysis of gene expression data from caArray. We first set up grid instances for caTissue [27] and caArray [28] holding clinical data [29] and experimental data (GEO GSE5949 [30]) on the NCI-60 cell lines. We then generated the OWL ontologies for caTissue, caArray, and the NCIt concepts they use from the metadata available from those services. The URLs for these ontologies are listed in Additional file 1. The ontologies were then loaded into BigOWLIM, a state-of-the-art semantic triple store. Extraction on caArray was performed by querying the caArray grid service and retrieving the transitive closure of objects related to the *Experiment* of interest. The same process was performed using the caTissue caGrid service for the *CollectionProtocol* of interest. The resulting data set included caArray *Source* s and caTissue *CellSpecimen* s. The results of those queries were then transformed into OWL Individuals that conformed to the OWL ontologies that were generated for caArray and caTissue. These data are available in N-Triples format in Additional files 2, 3, 4, 5. The data were then loaded into a KB with a custom inferencing rule that inferred the link between *Source* s and *CellSpecimen* s based on equivalence of the *CellLine* of the *Source* from caArray and the *Label* of the *CellSpecimen* in caTissue. Additional file 6 shows the inferencing rule. As a demonstration of the capability of this method we ran a query (Additional file 7) to extract the clinical diagnoses related to every *Hybridization* caArray. The results of the query are in Additional file 8. We used these diagnoses as labels in Figure 1, a Principal Components Analysis (PCA) Projection of GEO GSE5949 in caArray. The diagnoses that are shown are far more specific than the usual "cancer type" that is available in the GEO data set. Using this technique, other statistical analyses can be performed on annotations such as survival, gender, age at diagnosis, tissue site, or any number of clinical annotations that caTissue can be customized to contain.

The significance of this join is important: caArray and caTissue, while based on the same framework, share no application code and have been developed by different teams. Also, while the data are about the same biological entities, they were derived from separate sources at different institutions. A major benefit is that one can link and query data based on the NCIt concepts instead of the elements of the data model. This result shows the advantage of loading related data from unrelated caGrid services and link them using a semantic web framework. Performance of the repository is reasonable. Load times are shown in Table 1. We believe that SETL is a significant improvement over conventional ETL. One improvement is that this technique is not limited to a single data source. The rules developed apply to any caArray and caTissue data source, which makes cross-institutional integration easier. Another is that Semantic Web data warehousing allows a much cleaner separation of data into appropriate services. While caArray can store some of this information, it should not be considered a reliable source for information about biospecimens. Since microarray experiment records are static, only the information that is known at the time that the data is published and recorded is included in caArray. caTissue is designed to support the addition of more information (such as more specific diagnoses) about the biospecimens and sources as they become available. This is a promising development, as researchers can learn new information about existing experiments over time.

**Table 1:** Load and query performance. Load and query times for the operations used. The compute environment used an Intel Core 2 Quad @ 2.40 GHz and 4 GB of memory. The repository was single-threaded.

Stage	Data Size (Entities)	Data Size (Statements)	Processing Time (s)
**Loading Ontologies**	57,526	88,654	473
**Loading Data**	14.003	607,532	910
**Query**	-	-	3.24

Corvus is, at this time, still a prototype system with components that serve as proof of concept. We plan on expanding its ability in the future to allow for automated SETL and linkage of scientific data. Work also needs to be done on providing visualizations and other user interfaces.

## Conclusion

SETL is a valid technique for gathering information from semantically annotated grid services and using that semantic annotation as a means to search and view that information. It provides opportunities for integration of data that was not designed for that purpose. This allows for analysis of many different data types on a dynamic basis and makes it possible for informaticists to continually integrate relevant new data sources as they become available with far less effort than would be needed in a traditional data warehousing environment. Corvus, along with the caGrid security and semantic annotation infrastructure, allows for integration of data across institutions as well as across applications as long as those institutions use the same semantic metadata. This has large implications for increased collaboration in biomedical research.

## Methods

At the core of Corvus is a Semantic Web-based data warehouse based on BigOWLIM, within which we assemble our data by integrating various caGrid data sets. A key feature of our approach involves using OWL ontologies that have been generated from semantically annotated caGrid UML information models. Components of the Corvus framework support a Semantic ETL workflow that pulls data from public caGrid data services. It then translates that data into RDF/OWL that conforms to the OWL ontologies generated. Finally, it stores that information, along with the generated ontologies, in a Semantic Web KB. Because of this, it is possible to dynamically combine caGrid data sets while preserving semantic annotation of the caGrid information models. It also enables the use of Semantic Web technologies such as SPARQL (SPARQL Protocol and RDF Query Language), Semantic Web Rule Language (SWRL), and Description Logics (DL) reasoning services on that data.

Semantic ETL in Corvus consists of the following steps: generation of OWL ontologies from caGrid information models and loading them into the KB; submitting one or more queries to caGrid data services; transforming that data into RDF triples; and then loading those triples into the KB. As the data is loaded into the KB, custom rules are used to infer relationships between the data from the two sources. This allows queries to be joined through the inferred relationships.

### Data preparation

We use two caGrid database applications, caTissue and caArray, to demonstrate the ability to link related information from independent databases. caTissue is a biospecimen banking and management tool developed through the NCI for use in research tissue banks. It is able to store information about biospecimens and the individuals they originated from. caArray is a microarray management tool developed through the NCI and is a MicroArray and Gene Expression (MAGE)-compliant array repository. Both caTissue and caArray can publish data via caGrid services.

caTissue and caArray instances were deployed with caGrid services that published to the caGrid training grid. Expression data was loaded from GEO GSE5949 [30] by downloading the data and converting it into the MAGE-TAB format using the GEOImport and TabConverter tools from the tab2mage project [31].

Additional curation was needed to fix some references to array designs and to ensure that all *Characteristics [CellLine]* entries were valid and entered. The data was then uploaded to caArray [28]. Data on the cell lines, such as specific clinical diagnosis, was collected from the NCI SKY/M-FISH & CGH Database [29] and curated into a caTissue instance.

### Semantic ETL process

**Figure 2:**
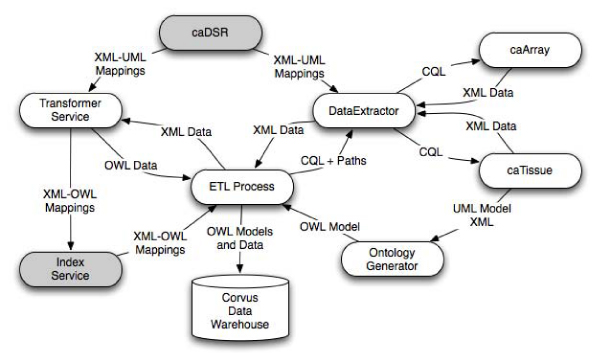
**Corvus ETL process**. The Corvus ETL Process.

The Corvus Semantic ETL process (Figure 2) is designed to enable new models and data to be dynamically integrated into the data warehouse. It is composed of the Ontology Generator, Data Extractor (Extraction), Data Transformer (Transformation), and finally a KB loader that loads the data into the data warehouse (Loading).

The Ontology Generator generates OWL ontologies from published caGrid data service UML information models. These ontologies represent the UML information model, semantic annotations on those models, and the relevant parts of the NCIt. We generated ontologies from the caArray 2.1 and caTissue Suite 1.1 models. These ontologies are then loaded into the Corvus data warehouse.

The Data Extractor handles CQL queries of objects and the relationships between those objects. For example, we query caTissue for a *CollectionProtocol* object. Here, the path information indicates how the associated *CellSpecimen* objects should be included in the resulting object graph. The Data Extractor uses the CQL and path information to pull XML data from caGrid data services. The ETL Process then passes the XML data to a Transformer Service instance that provides an XML to OWL transformation. The resulting OWL instance data is then loaded in the Corvus data warehouse.

#### Ontology generator

**Figure 3:**
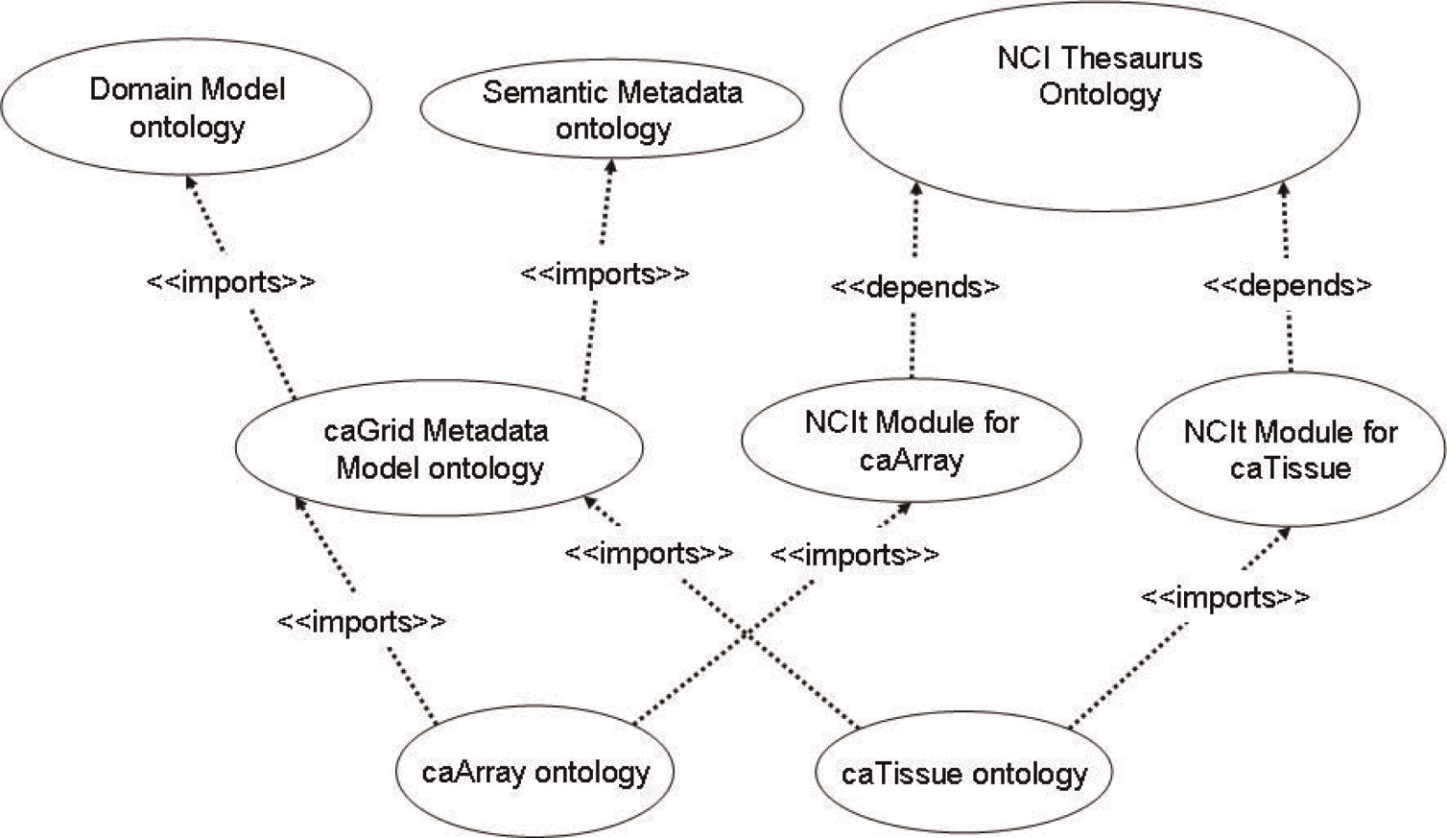
**Modules diagram**. Package diagram showing the import and dependency relationships between ontology modules.

We implemented an automatic transformation process from grid services metadata (annotated UML models) into OWL ontologies. In order for this process to be successful, it is necessary to first model the structure of the caGrid metadata in OWL. We decided to build two helper ontologies for this task: the Domain Model ontology and the Semantic Metadata ontology. The Domain Model ontology represents the relationships that exist among UML entities, including class-attribute relationships and relationships between classes. The Semantic Metadata ontology, on the other hand, represents relationships between caGrid entities and NCIt concepts. These two helper ontologies are imported during the UML to OWL transformation process, and are the same for any UML-derived OWL ontology. Figure 3 shows the import trail of these ontologies into the generated caArray and caTissue ontologies. As can be seen, we take a modular approach, where we merge the Semantic Metadata ontology and the Domain Model ontology into a caGrid Metadata ontology before importing them into the generated OWL ontologies. The Semantic Metadata ontology models the NCIt annotations of UML classes and attributes using the object property *semanticMetadataCollection*. The Domain Model ontology represents UML class-attribute relationships using the *umlAttributeCollection* object property and relationships among UML classes are modeled as children (sub-properties) of the object property *umlAssociation*.

We also need a third helper ontology to represent the NCIt concepts relevant to a particular caGrid UML model. While we could import the whole (OWL-transformed) NCIt, we were interested in extracting the relevant NCIt concepts to reduce the overall ontology size. Figure 3 shows that the resulting ontologies are specific for a particular caGrid model, such as caTissue (NCIt Module for caTissue) and caArray (NCIt Module for caArray). We use the methodology in [32] to extract relevant subsets from NCIt. This methodology has the following properties [32]: a) it preserves NCIt semantics; b) it includes everything that is relevant to the particular information model ontology; and c) it imports only what is relevant. The resulting NCIt Module ontologies are then imported during the UML-to-OWL transformation process (Figure 3).

**Figure 4:**
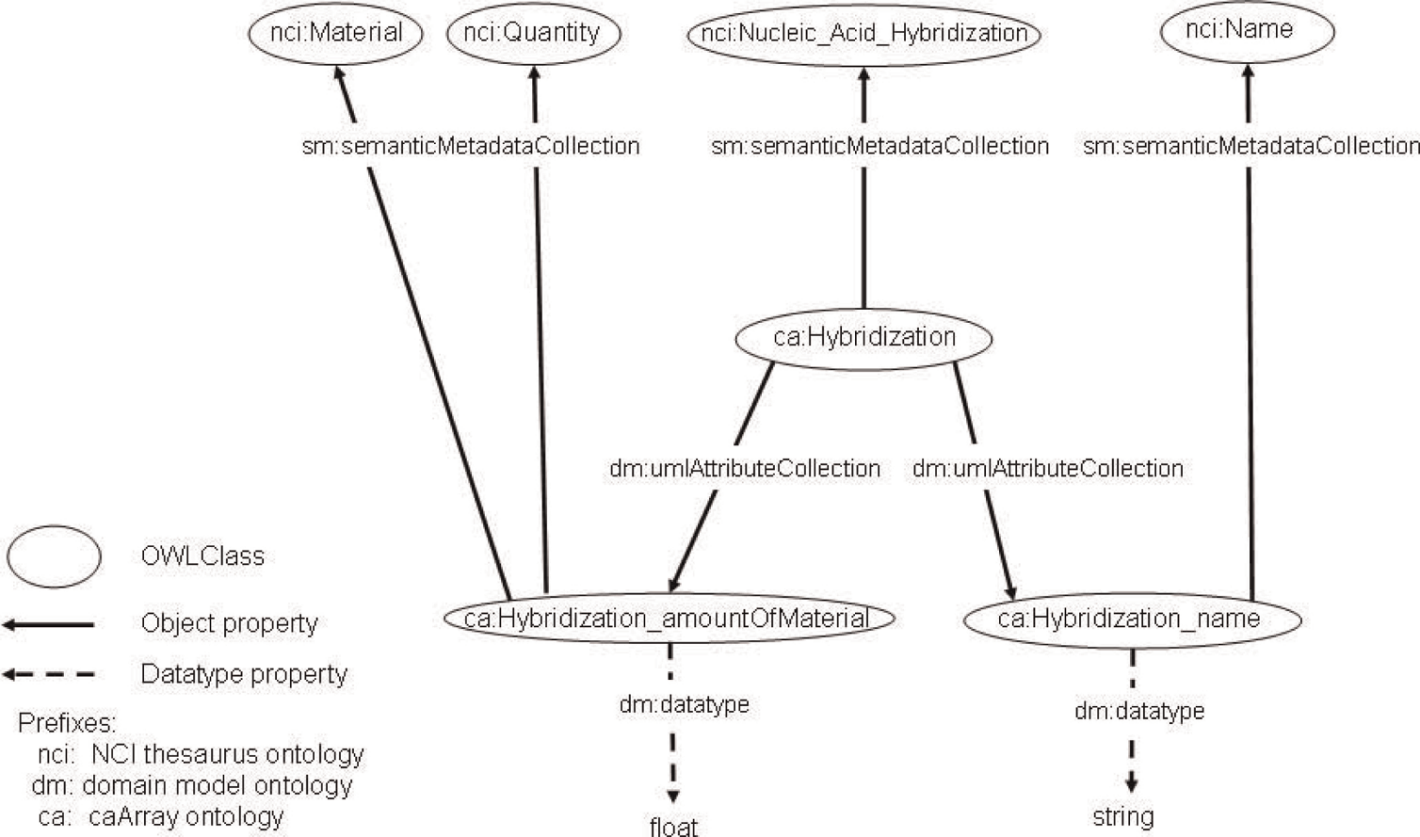
**Annotated UML class OWL representation**. Representation of the Hybridization class from caArray in OWL format. *Hybridization* is annotated with the NCIt term "Nucleic Acid Hybridization" and has two attributes: "name" and "amountOfMaterial", which in turn have their NCIt annotations. The values of these attributes are maintained via the *dm:datatype* property.

Figure 4 explains how the three helper ontologies facilitate the UML-to-OWL transformation, depicting part of the caArray 2.1 generated ontology. OWL-transformed UML classes are associated with NCIt concepts via the object property *semanticMetadataCollection*, such as *ca:Hybridization* with the NCIt concept *nci:Nucleic_Acid_Hybridization*. In order to keep the ontologies decidable (OWL-DL), we do not assert relationships by this property. Rather, the OWL-transformed UML classes are defined as sub classes of an existential restriction on the *semanticMetadataCollection* property, where that property has some values from a class defined in NCIt. UML attributes are individuals rather than properties. Their OWL-transformed UML class links to them via the object property *umlAttributeCollection*, such as *ca:Hybridization* with *ca:Hybridization name*. Attributes have NCIt annotations themselves, linked via the object property *semanticMetadataCollection*. In addition, attributes have a datatype property *dm:datatype* that contains the value of that attribute. There are further characteristics of our UML-to-OWL transformation strategy. UML classes and UML attributes are defined as subclasses of *dm:UMLClass* and *dm:UMLAttribute*, respectively. UML class hierarchies are represented with the *rdfs:subClassOf* construct. UML class associations (*a has_a b*) are modeled as *rdfs:subPropertyOf umlAssociation*. We use OWL cardinality restrictions to represent multiplicities of UML associations. Also, if an association is bidirectional, an inverse property is defined. UML classes and attributes are defined as disjoint unless they have a relationship in the class hierarchy. Since the DL expressiveness of the resulting ontologies is (*D*) [18], it is possible to use existing OWL-DL reasoners with them. The generated ontologies provide an integrated view and formal representation of the caGrid data services' metadata. As shown below, these ontologies can be extended to consider instance data, providing the semantic framework for data integration.

#### Data extractor

The Data Extractor component works with most caGrid data services that have been generated from the caCORE Software Development Kit (SDK). For this effort, we queried caArray and caTissue. The Data Extractor relies on knowledge of caCORE conventions for naming of object identifiers (i.e. primary keys) and XML-UML mapping rules. A future enhancement to the Data Extractor may pull metadata about XML-UML mapping rules and identifiers directly from caDSR.

**Figure 5:**
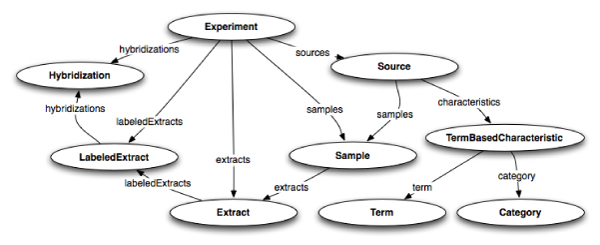
**caArray query paths**. caArray query paths – this graph is needed to extract all information about an experiment.

Since the current version of CQL does not support projections and the XML results returned by most caGrid data sources do not contain foreign key values, we cannot avoid what is know as the "*n* + 1 select" problem [33], in which we must execute one query to retrieve an initial data set and then *n* additional queries to retrieve information associated to each item in the initial data set, where *n* is the size of the initial data set. Some data services, such as caArray, partially address this problem by automatically including information about associated objects in the XML results document. For example, the result of a query for *Experiment* s includes information about all *Hybridization*, *Sample*, *Source*, *Extract*, and other objects that are associated with each *Experiment*. The next version of CQL will allow the query to indicate what associated objects should be included in the results [34]. It took about 20 minutes to extract the data we need from a caArray *Experiment* containing 300 *Hybridization* s. Figure 5 depicts the paths that were traversed.

#### Transformer services

We expose an XML to RDF/OWL Transformation service to convert caGrid XML to RDF that conforms to the ontologies generated by the ontology generator. The Transformer Service is a generic service that exposes any configured XML-to-XML transformation (including XML to RDF/OWL) as a stateful grid service. These services advertise what kinds of transformations they support and therefore enable clients to dynamically discover available transformations. We have provided a general-purpose Transformer implementation that will transform XML from caCORE SDK generated data services by using caCORE SDK UML-to-XML conventions. A future enhancement may pull UML-to-XML mapping metadata from caDSR.

#### Loading data from caTissue and caArray

The output from the transformation service was then loaded into Corvus. Corvus supports a number of triple stores, but in this case we used BigOWLIM. We had two sets of transformed data: data from caTissue and data from caArray. To link the two data sets, we make use of the caTissue and caArray data models stored in Corvus and write a rule that links the *Source* (biological source) object in caArray to the *CellSpecimen* object in caTissue if *Source.CellLine* and *CellSpecimen.Label* are equivalent. Inferencing was done using a custom rule implemented in the Ontotext's TRREE language, used by BigOWLIM.

Additional file 6 shows the actual rule, which adds the triple *Source derived from CellSpecimen* to the store. The inverse triple, *CellSpecimen derived_by Source* is also added.

### Data queries and analysis

The query in Additional file 7 returns the caTissue clinical diagnosis using the NCIt concept "Clinical Diagnosis" and the name of the caArray *Hybridization* it corresponds to. Also available, but not extracted, are: gender, age at diagnosis, ethnicity/race, or any other clinical annotations that are added to a caTissue Suite repository. A Principal Components Analysis is made of the expression data using the PCA module from GenePattern [35] and the projection is colored with the diagnoses extracted.

## Electronic supplementary material

Additional file 1: List of external ontology URLs. (LIST 586 bytes)

Additional file 2: N-Triple data for caTissue *CollectionProtocol*, *Participant*, and *Specimen* individuals. (NTP 1 MB)

Additional file 3: caArray individual Linkages in N-Triple format. (NTP 2 MB)

Additional file 4: caArray *Experiment* Data and *Annotation* s in N-Triple format. (NTP 2 MB)

Additional file 5: caArray *SourceCharacteristic* s in N-Triple format. (NTP 6 MB)

Additional file 6: A TRREE inferencing rule that describes an inferred relationship between *CellSpecimen* s in caTissue and *Source* s in caArray. The relationship here is derivation, as described by the *derived_from* and *derived_by* properties. This rule finds all caArray *Sources* that have a *TermBasedCharacteristic* where the *Term value* matches the *Label* of a caTissue *CellSpecimen*. The *Label* used is found through the NCIt term *nci:Label*. (PIE 1 KB)

Additional file 7: SPARQL query used to retrieve the clinical diagnosis for the cell lines used in GEO GSE5949. The query takes advantage of the inferred relationship *derived_from* that is described in Additional file 6. (SPARQL 2 KB)

Additional file 8: Diagnoses for hybridizations, tab separated. (TXT 13 KB)
